# When Field Experiments Yield Unexpected Results: Lessons Learned from Measuring Selection in White Sands Lizards

**DOI:** 10.1371/journal.pone.0118560

**Published:** 2015-02-25

**Authors:** Kayla M. Hardwick, Luke J. Harmon, Scott D. Hardwick, Erica Bree Rosenblum

**Affiliations:** 1 Department of Biological Sciences, University of Idaho, Moscow, Idaho, United States of America; 2 Department of Mechanical Engineering, University of Idaho, Moscow, Idaho, United States of America; 3 Department of Environmental Science, Policy, and Management, University of California, Berkeley, California, United States of America; University of Calgary, CANADA

## Abstract

Determining the adaptive significance of phenotypic traits is key for understanding evolution and diversification in natural populations. However, evolutionary biologists have an incomplete understanding of how specific traits affect fitness in most populations. The White Sands system provides an opportunity to study the adaptive significance of traits in an experimental context. Blanched color evolved recently in three species of lizards inhabiting the gypsum dunes of White Sands and is likely an adaptation to avoid predation. To determine whether there is a relationship between color and susceptibility to predation in White Sands lizards, we conducted enclosure experiments, quantifying survivorship of *Holbrookia maculate* exhibiting substrate-matched and substrate-mismatched phenotypes. Lizards in our study experienced strong predation. Color did not have a significant effect on survival, but we found several unexpected relationships including variation in predation over small spatial and temporal scales. In addition, we detected a marginally significant interaction between sex and color, suggesting selection for substrate matching may be stronger for males than females. We use our results as a case study to examine six major challenges frequently encountered in field-based studies of natural selection, and suggest that insight into the complexities of selection often results when experiments turn out differently than expected.

## Introduction

Natural selection plays a central role in shaping patterns of diversification in natural populations [1–4], and is thus a major focus of the field of evolutionary biology. In order to understand how adaptation to distinct environments can result in population differentiation and ultimately speciation, researchers must accurately identify phenotypic targets of divergent natural selection [5]. Trait differences between populations are often assumed to have evolved via natural selection [6], representing initial stages of ecological speciation. However, direct evidence of specific traits conferring a fitness advantage to locally adapted individuals in natural populations is difficult to obtain and thus somewhat uncommon (e.g., [7], but see [8]). As a result, evolutionary biologists currently have an incomplete understanding of the adaptive significance of specific traits in most systems.

White Sands is an ideal system for taking an experimental approach to understanding how selection on specific traits facilitates adaptation to distinct environments. The area is characterized by white gypsum sand dunes that formed less than 10,000 years ago [9]. In contrast, the surrounding “dark soils” desert scrubland is characterized by brown substrate. Three lizard species are found in both the dark soils and White Sands habitats: *Holbrookia maculata* (the Lesser Earless Lizard), *Sceloporus cowlesi* (the Eastern Fence Lizard), and *Aspidoscelis inornata* (the Little Striped Whiptail). For all three species of lizards, populations in dark soils exhibit brown dorsal color, while populations in White Sands have blanched dorsal color [10–13]. The evolution of blanched color in White Sands is most likely an adaptation to avoid detection by visually oriented avian predators such as *Lanius ludovicianus* (the loggerhead shrike) and *Geococcyx californianus* (the greater roadrunner), both of which occur in White Sands [14–16].

A number of lines of evidence indicate that blanched dorsal color is adaptive in White Sands lizards. First, there is a correlation between color phenotype and the substrate environment, despite ongoing gene flow [17]. Second, there is convergence among multiple taxa in the evolution of blanched color in White Sands populations [18]. A number of vertebrate and invertebrate species exhibit blanched color in White Sands populations and a darker dorsal phenotype in dark soils populations, including the three species of lizards mentioned above as well as *Perognathus gypsi* (the Apache pocket mouse) [19], *Scaphiopus couchii* (Couch’s Spadefoot Toad) [20], and *Daihinoides hastiferum* (the White Sands camel cricket) [21]. Third, analyses of molecular data suggest that *Mc1r*, which plays a role in determining the density and distribution of melanin in the skin of vertebrates [22], has been under divergent selection in this system. There is a significant correlation between the blanched color phenotype and alleles at the *Mc1r* locus in all three White Sands lizard species [23], and in two of the three lizard species (*S. cowlesi* and *A. inornata*) the functional basis of pigmentation loss via specific *Mc1r* mutations has been identified [24]. Genetic divergence between White Sands and dark soils lizard populations is greater at the *Mc1r* locus than at neutral loci [23], indicating that selection on body color has played a role in local adaptation in this system.

In light of previous research suggesting there has been selection on dorsal color at White Sands, we sought to experimentally investigate the adaptive significance of blanched color. Specifically, the objective of our study was to determine whether there is a relationship between body color and susceptibility to predation in White Sands *H. maculata*. We conducted enclosure experiments with *H. maculata* exhibiting substrate-matched and substrate-mismatched color phenotypes (i.e., phenotypes exhibited by White Sands and dark soils lizards, respectively) and quantified survivorship from predation. We report results from our primary objective (assessing selection on color), and also report several unexpected findings that suggest selection at White Sands may be more complex than previously considered.

## Materials and Methods

### Study Design

Animal care and use protocols for all experiments were approved by the University of California Berkeley ACUC (R347) and the University of Idaho IACUC (2009–37), and permits for field work were approved by White Sands National Monument, White Sands Missile Range, and the New Mexico Department of Game and Fish. We performed enclosure experiments with *H. maculata* individuals, because *H. maculata* exhibit the highest degree of divergence in body color between White Sands and dark soils individuals of the three White Sands lizard species [25]. In addition, they are likely the most reliant on substrate matching of the three species. *H. maculata* are sit and wait predators (while *A. inornata* are active foragers [26]) and are less closely associated with vegetation than *S. cowlesi* [13]. *Holbrookia maculata* individuals in White Sands are active during the months of May through October from 0700 to 1900 hours (with peaks in activity occurring daily from 1000 to 1400 hours) [13], and we conducted trials from May through July in 2011 and 2012.

In 2011 we built three enclosures on the White Sands ecotone, each 100 square meters (10 meters by 10 meters). The ecotone is a narrow band of transitional habitat at the edge of the White Sands formation with light gypsum substrate but a higher density of vegetation than is found in the heart of the dunes. The ecotone was the optimal location to conduct our study because predator densities are also higher in the ecotone than in the heart of the dunes [27], and because ecotone *H. maculata* are blanched and indistinguishable in color from those found in the heart of the dunes [25]. While the substrate color of the ecotone is more variable [25], we built enclosures in regions where sand color was comparable to that of the heart of the dunes. The mean distance between enclosures was 113.23 meters.

**Fig 1:**
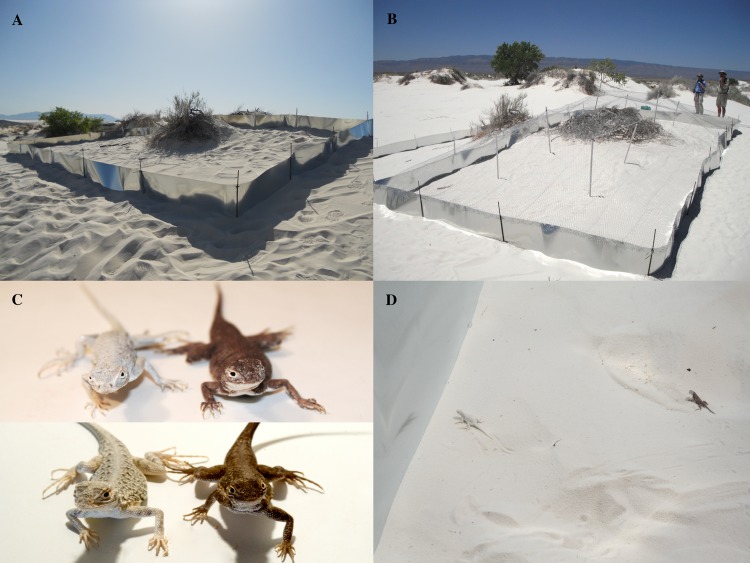
Design of the enclosure experiment. Panel A shows the “open” enclosure treatment, which allowed avian predators to enter and exit freely, and panel B shows the “closed” enclosure treatment, which excluded avian predators with chicken wire. The top half of panel C shows color-manipulated *H. maculata* (with the substrate-matched paint treatment on the left and substrate-mismatched paint treatment on the right), and the bottom half shows the corresponding naturally occurring color phenotypes (with the White Sands phenotype on the left and dark soils phenotype on the right) (photograph courtesy of S. Des Roches). Panel D shows a substrate-matched lizard (left) and a substrate-mismatched lizard (right) after release into an enclosure.

We constructed the enclosures using steel flashing (0.5 meters in height with approximately 15 centimeters buried under the surface of the sand). To anchor flashing in place we fastened it to rebar posts (1.2 meters in height with approximately half of each post buried under the surface) using zip ties. We divided each enclosure in half with a 10 meter long piece of flashing, and covered seams using aluminum foil tape. In 2011, one half of each enclosure allowed avian predators to enter freely (the “open” treatment), and we covered the other half with chicken wire to exclude predators (the “closed” treatment) (Fig. 1). To create the closed treatment, we used zip ties to fasten chicken wire to the top edge of the steel flashing for one half of each enclosure. To ensure lizards would have an ongoing supply of food we used chicken wire with holes 2.54 centimeters in diameter, which allowed invertebrates to enter and exit freely. To support the chicken wire we placed a number of rebar posts throughout the closed sides of enclosures, and covered support posts with PVC pipe so that the lizards could not use supports to escape. To make sure lizards had adequate ground cover to seek refuge from biotic and abiotic elements of the environment, we chose enclosure locations where existing vegetation covered approximately 20% of the available space (previous research indicates that White Sands *H. maculata* prefer habitat where on average 20.5% of the ground is covered by vegetation [13]). We ensured that our enclosure design was sufficient to contain lizards by conducting brief (15 minute) observations of *H. maculata* within enclosures before initiating trials.

We captured *H. maculata* individuals by hand or by noose from several geographically proximate locations throughout White Sands National Monument (with a maximum distance of nine kilometers between locations) to ensure that we did not impact any one subpopulation disproportionately. Our prior studies have not shown differentiation in behavior, morphology, or genetics among sampling localities within White Sands [28]. To control for any subtle differences among lizards collected from different localities, we randomly assigned lizards to experimental treatments. We recorded sex and took measurements of mass, snout-vent length, and tail length for all individuals. Morphological data for individual lizards are available in the Dryad repository (doi: 10.5061/dryad.068b6). We included adult and subadult lizards in our trials in a body size range of 3.4 to 6.1 centimeters snout-vent length. We excluded juvenile lizards given the potential for predation by larger conspecifics during the course of the experiment. We randomly assigned lizards of different sizes to different treatments and enclosures. We used a black permanent marker to record an individual identification number on the ventral surface of each lizard. Before and after trials, we housed lizards individually in small cages with 12-hour light cycles. We kept lizards in captivity up to 10 days before the beginning of trials, and released them at their original points of capture no more than one week after trials were completed. While lizards were in captivity we fed them *ad libitum*.

To test whether dorsal color had an effect on lizard survivorship in White Sands, we painted *H. maculata* dorsal surfaces to be substrate-matched or substrate-mismatched (i.e., to represent the color of White Sands or dark soils lizards, respectively) using human temporary tattoo paint (Amunez International) (Fig. 1). Previous research has shown that tattoo paint can be used to alter the color of lizards for extended periods of time without harmful effects [29]. We chose to use painted lizards in our experiment rather than lizards captured from dark soils because we were interested in specifically investigating the role of body color in survival. Previous research on the White Sands system has shown that lizards in White Sands differ from those in dark soils in a number of characteristics besides color, including aspects of morphology and behavior [27, 30–31]. By using lizards exclusively from White Sands, we controlled for potentially confounding characteristics that differ between populations and were able to examine the effects of color specifically; we were also able to avoid unintentionally releasing non-native individuals into White Sands populations and prevent breeding between White Sands and dark soils individuals.

To obtain paint colors that corresponded to White Sands and dark soils dorsal color, we used spectrometer readings of *H. maculata* dorsal surfaces taken during current and previous field seasons (n = 49 *H. maculata* from White Sands, and n = 23 *H. maculata* from dark soils) [25]. We determined for both the substrate-matched and substrate-mismatched phenotypes the ratios of white, black, and brown paints that, when mixed and applied to the dorsal surfaces of White Sands lizards, produced absorbance curves where spectrometer readings fell within the minimum and maximum absorbance values observed at each wavelength from 300–800 nanometers (which encompasses the range of the spectrum visible to most birds [32]) for White Sands and dark soils lizards. Finally, we painted all lizards to be used in enclosure trials, randomly assigning each individual to either the substrate-matched or substrate-mismatched paint treatment. We took digital photographs of dorsal surfaces before and after painting for all individuals. In addition, for a subset of individuals (n = 24 from the substrate-matched treatment and n = 25 from the substrate-mismatched treatment) we took measurements of dorsal and ventral coloration before and after painting using a StellarNet EPP2000Cs spectrometer (StellarNet,Tampa, Florida; UV-VIS range of 280–900 nanometers) with a deuterium and tungsten/halogen light source (SL4-DT) and a reflectance probe (R600–8-UV-VIS-SR) fitted with a 45 degree angle tip (RTIP45). Spectrometer data for painted and unpainted lizards collected in 2011 and 2012 are available in the Dryad repository (doi: 10.5061/dryad.068b6).

In order to ensure that the paint treatments did not interfere with the ability of lizards in the study to thermoregulate, we assessed thermal preference using 20 White Sands *H. maculata*. Half of the individuals used to measure thermal preference were painted to be substrate-matched, and the other half were painted to be substrate-mismatched. Equal proportions of males and females were used in each paint treatment group. To assess preference we filled a 37.9 liter terrarium with White Sands substrate. We placed a light source at one end so that a gradient of substrate temperatures ranging from 27 to 45 degrees Celsius was available within the terrarium. We housed each lizard at room temperature without a heat lamp immediately prior to assessing thermal preference, and then placed the lizard in the center of the terrarium and allowed it to acclimate for 30 minutes. We took measurements of temperature with a cloacal probe at 30 minute intervals over a 90 minute period, resulting in data collection points at 30, 60, and 90 minutes. At each time point we also recorded the distance of the lizard from the terrarium light source. Thermal preference data are available in the Dryad repository (doi: 10.5061/dryad.068b6). We collected data at multiple time points to ensure that lizards in different paint treatments did not differ in their ability to maintain preferred body temperature over time. We performed assessments of thermal preference on May 31, 2011 and June 1, 2011.

After processing and painting, we released lizards into enclosures (Fig. 1). We gave all lizards a substantial meal (two medium crickets or mealworms) the evening prior to being released into enclosures. Immediately before initiating trials, we ensured enclosures were empty of lizards and other vertebrates by checking visually and thoroughly raking the sand. We included 14 lizards in each half enclosure, seven from the substrate-matched treatment group and seven from the substrate-mismatched treatment group. We assigned individuals randomly to enclosure treatment, while ensuring that the proportions of males and females in matched and mismatched treatments were consistent across open and closed sides of each enclosure. In 2011, we included all lizards from the thermal preference assessment in a single enclosure trial and randomly assigned them to the open or closed treatment group.

We started the first enclosure trial on June 1, 2011, and staggered start dates for subsequent trials by several days each. By staggering start dates, we were able to increase replication without building additional enclosures, and were also able to run trials over a greater proportion of the activity season. Once a trial was initiated, we visited enclosures frequently (once every two to three days, or up to three times a day in inclement weather) to ensure structural integrity of the enclosures. During trials we also checked enclosures every several days for signs of visitation by possible predators (i.e., tracks, scat, feathers, owl pellets, hair, shed skin, or lizard carcasses). During enclosure checks we removed any individuals that had shed their skin and transported them to the field station to repaint them. We released repainted individuals into their original enclosures within 24 hours of removing them. We captured survivors from both open and closed sides of enclosures after 16 days. At the end of trials, we recorded the mass of surviving lizards and checked for instances of tail autotomy.

For trials in 2012 we used a total of four enclosures, which included the three constructed in 2011, plus an additional enclosure built in a nearby location in the White Sands ecotone in May 2012. Because we had 100% survivorship of lizards in the control treatment in 2011 (see Results), we did not repeat the predator exclusion portion of the study in 2012. We removed chicken wire from all previously built units so that we had four 100 square meter enclosures, each divided in half for a total of eight open treatment replicates. We included 14 lizards in each half enclosure, seven from the substrate-matched treatment group and seven from the substrate-mismatched treatment group. We staggered start dates for enclosures by several days each, with the earliest starting on May 30. For two enclosures we performed an additional round of enclosure trials after recapturing the first set of survivors, using the same methods with new lizards. We ran a total of three open treatment replicates (n = 42 lizards) and three closed treatment replicates (n = 42 lizards) among three enclosures in 2011, and in 2012 we ran a total of 12 open treatment replicates (n = 168 lizards) among four enclosures. Thus the total number of open treatment replicates for 2011 and 2012 together was 15 (n = 210 lizards). Recapture data for individual enclosure replicates are available in the Dryad repository (doi: 10.5061/dryad.068b6).

### Statistical Analysis

To evaluate how well paint color treatments corresponded to the natural color of White Sands and dark soils lizards, we compared the brightness of painted lizards to that of the White Sands and dark soils lizards used to generate the paint treatments. Brightness is the component of color that accounts for 90% of variation between *H. maculata* from White Sands and dark soils [25], and is therefore a good indication of the degree to which paint treatments represent the color of lizards from the two distinct habitats. We used Endler’s segmentation method [33] to measure brightness over the wavelength range of 300–800 nm for painted and unpainted lizards, and then compared brightness of White Sands and dark soils lizards to that of the corresponding paint treatment using t-tests. To determine whether paint treatment differentially affected the ability of lizards to thermoregulate, we performed ANCOVAs comparing both body temperature and distance from the light source between paint treatments, with time as a covariate.

To understand the dynamics of predation in our enclosure trials, we compared survivorship of lizards with respect to enclosure treatment and paint color treatment. We used Fisher’s exact tests to compare the number of surviving lizards between open and closed enclosure treatments for trials conducted in 2011. In addition, we used chi-square contingency tests to compare (within open enclosures) the number of surviving lizards in substrate-matched and substrate-mismatched paint color treatments for trials from 2011 and 2012. Finally, we fit a general linear model with a binomial link function to the open enclosure data to test the effects of paint treatment, enclosure location, and year on survivorship. Specifically, our response variable was lizard survivorship (yes or no), and our explanatory variables were paint treatment (matched or mismatched), enclosure (one of four possible geographic locations within the ecotone), year (2011 or 2012), sex, and snout-vent length. In addition, we included pairwise interactions between each of these terms in our model. Including enclosure as a factor in our model allowed us to account for the fact that replicates in the same enclosure experience the same microhabitat. We conducted all statistical analyses in R version 2.15.2 [34].

## Results

Paint treatments accurately reflected natural colors and did not differentially impact the ability of lizards to thermoregulate. Lizards from the substrate-matched paint treatment did not differ significantly from White Sands *H. maculata* in dorsal brightness (t_45.79_ = -1.61, P = 0.11), and the same was true for substrate-mismatched lizards and dark soils *H. maculata* (t_45.99_ = -1.15, P = 0.25). Substrate-matched lizards were significantly brighter than substrate-mismatched lizards (t_45.79_ = 16.41, P < 0.01), which is consistent with differences between the naturally occurring White Sands and dark soils phenotypes. In addition, paint treatment groups did not differ significantly in average body temperature (F_1, 333_ = 0.0, P = 1.0) or distance from the light source (F_1, 11928_ = 0.13, P = 0.72), and these patterns were consistent over the course of the entire trial time period (P = 0.33 for body temperature; P = 0.50 for distance from light source). Lizards utilized in the thermal preference portion of the study did not suffer decreased survivorship in the enclosure trials; survivorship in the open side of the enclosure with thermal preference lizards in 2011 was 43%, while survivorship in replicates in the same enclosure in 2012 was 41% (95% CI [0%, 85%]).

**Fig 2:**
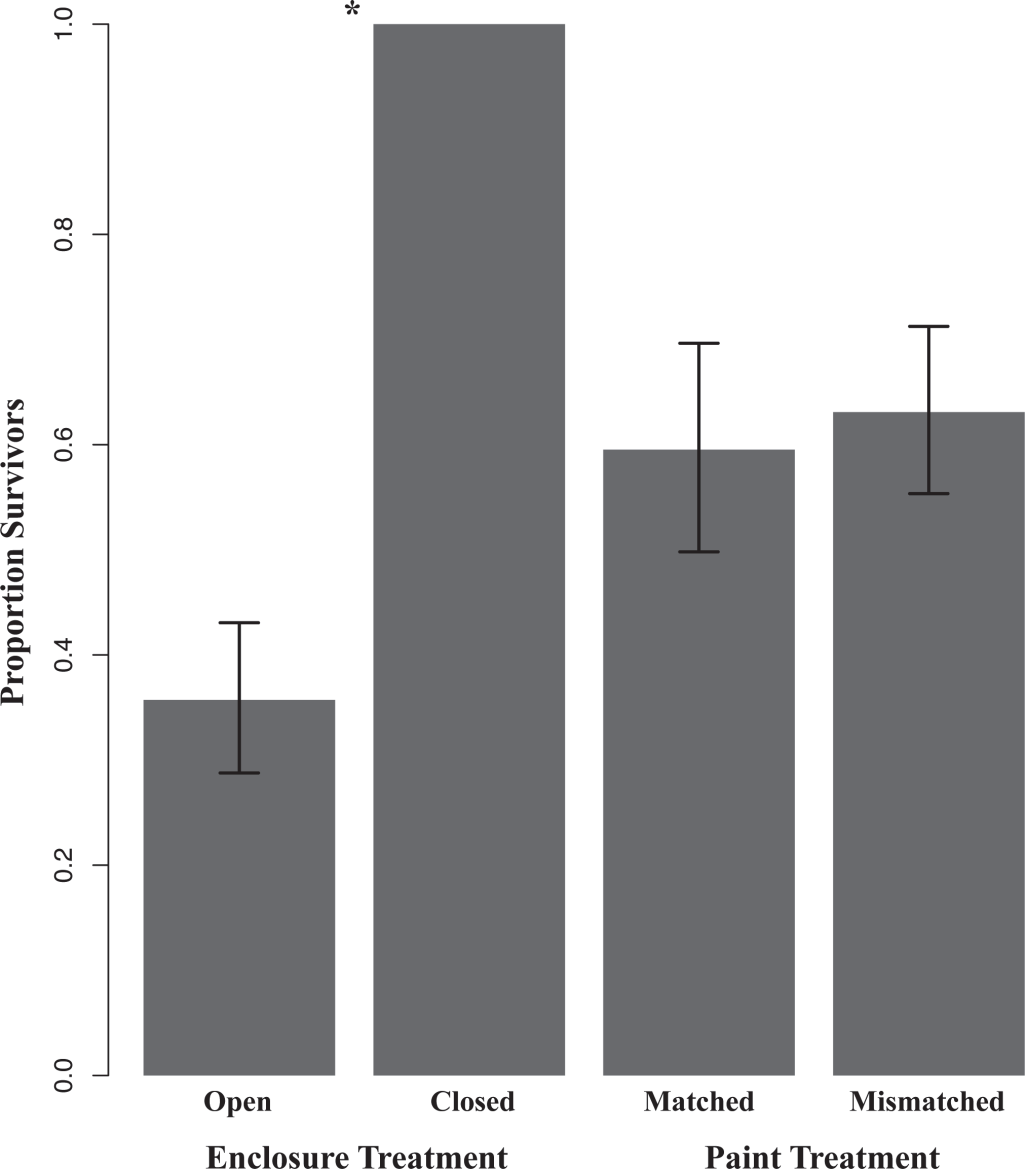
Proportion of lizards that survived trials with respect to enclosure treatment and paint treatment. Survivorship was significantly lower in the open enclosure treatment than in the closed enclosure treatment (P < 0.05 indicated by an “*”). We did not detect a significant difference in survivorship by paint treatment group. Bars represent mean survivorship across enclosure replicates, and vertical lines indicate the standard error of the mean. The open versus closed enclosure treatment comparison includes data from 2011 trials only (three open and three closed replicates, n = 84 lizards). The matched versus mismatched paint treatment comparison includes data from 2011 and 2012 trials (15 open replicates, n = 210 lizards).

Survivorship differed significantly between open and closed treatments in trials conducted in 2011, where survivorship was higher for lizards in closed treatments than in open (Fisher’s exact test, P < 0.01). In fact, 100% of the lizards within the closed treatment group survived, compared with only 36% (95% CI [5%, 67%]) of lizards in the open treatment group (Fig. 2). However, survivorship in open replicates did not differ between paint treatments (χ^2^_1_ = 0.02, P = 0.89). 36% of lizards in the open treatment survived in 2011, with 38% (95% CI [0%, 100%]) of substrate-matched lizards surviving, and 33% (95% CI [13%, 53%]) of substrate-mismatched lizards surviving. In 2012, 61% (95% CI [42%, 80%]) of lizards survived, with 59% (95% CI [37%, 81%]) of substrate-matched lizards surviving, and 63% (95% CI [46%, 80%]) of substrate-mismatched lizards surviving. Thus for 2011 and 2012 combined, 56% (95% CI [40%, 72%]) of all lizards in the open treatment survived, with 55% (95% CI [36%, 74%]) of substrate-matched lizards surviving and 57% (95% CI [42%, 72%]) of substrate-mismatched lizards surviving (Fig. 2).

**Fig 3:**
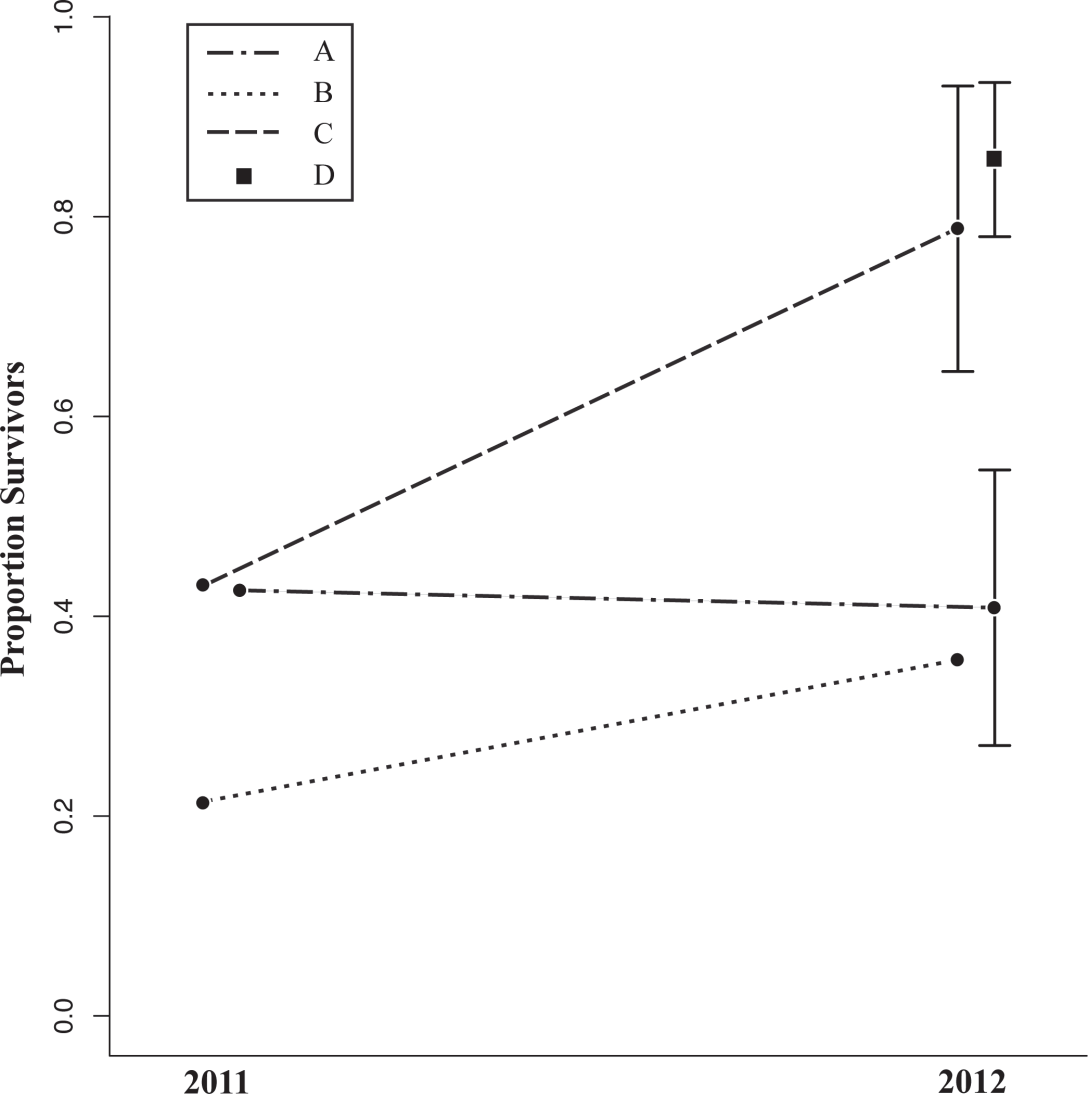
Proportion of lizards that survived trials with respect to year and enclosure location. Survivorship differed significantly between years (P = 0.03) and among enclosures (P < 0.01) in our general linear model. In addition, we detected an interaction between year and enclosure (P = 0.03), where survivorship in some enclosures differed significantly between years and survivorship in other enclosures did not. Dashed lines represent the interaction effect between year and enclosure on survivorship that we detected in our linear model, with endpoints representing mean proportion of survivors in replicates within different enclosures in different years. Solid, vertical lines indicate the standard error of the mean for enclosures in years with multiple replicates, where survivorship varied among replicates. We used open replicate data from each year to calculate survivorship for each enclosure (one replicate in 2011 and four in 2012 for enclosure A [n = 70 lizards]; one in 2011 and two in 2012 for enclosures B and C [n = 42 lizards each]; zero in 2011 and four in 2012 for enclosure D [n = 56 lizards]).

**Fig 4:**
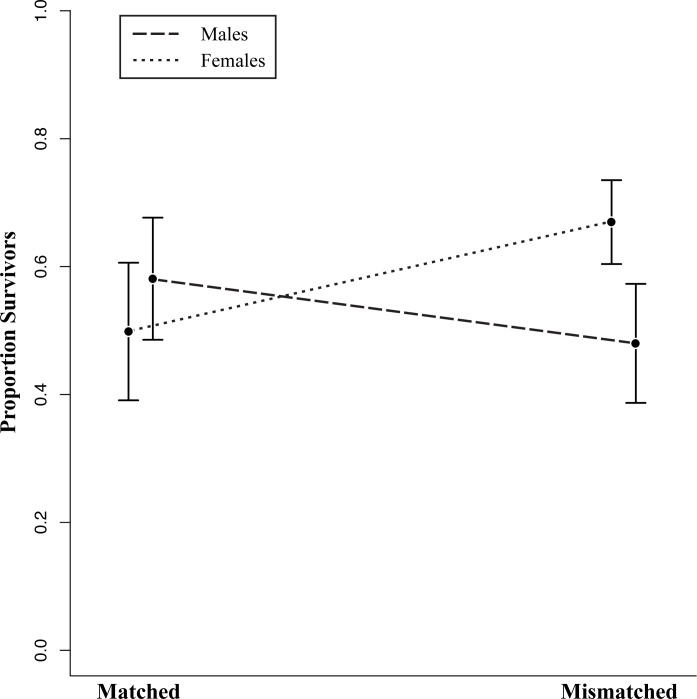
Proportion of lizards that survived trials with respect to paint treatment group and sex. We detected a marginally significant trend between sex and paint treatment in our general linear model (P = 0.05), where substrate-matched males had higher survivorship than substrate-mismatched males, but substrate-matched females had a lower survivorship than substrate-mismatched females. Dashed lines represent the interaction effect between sex and paint treatment on survivorship that we detected in our linear model, with endpoints representing mean proportion of survivors of each sex in different paint treatments across enclosure replicates. Solid, vertical lines indicate the standard error of the mean. We used open replicate data from 2011 and 2012 to calculate survivorship for each category (for a total of 15 open replicates, n = 210 lizards).

The results of our general linear model indicated that lizard survivorship did not vary with respect to paint treatment (χ^2^_1_ = 0.037, P = 0.85), but did vary with respect to enclosure location and year. Survivorship was significantly higher in trials in 2012 compared with trials in 2011 (χ^2^_1_ = 4.46, P = 0.03) (Fig. 3). In addition, survivorship for trials in 2011 and 2012 differed dramatically among enclosures (χ^2^_3_ = 37.58, P < 0.01); two enclosures exhibited higher survivorship with 67% (95% CI [5%, 100%]) and 86% (95% CI [62%, 100%]) of lizards recaptured, and two enclosures exhibited lower survivorship with 41% (95% CI [11%, 71%]) and 31% (95% CI [11%, 51%]) of lizards recaptured (Fig. 3). Our model detected a significant interaction between enclosure and year (χ^2^_2_ = 7.11, P = 0.03), where survivorship in some enclosures differed significantly between years and survivorship in other enclosures did not (Fig. 3). We also observed a marginally significant interaction between paint treatment and sex (χ^2^_1_ = 3.64, P = 0.05). Mean survivorship of mismatched males was 48% (95% CI [28%, 68%]), and survivorship of matched males was 58% (95% CI [38%, 78%]). The opposite pattern occurred in females, with 67% (95% CI [53%, 81%]) of mismatched individuals surviving, compared with 50% (95% CI [27%, 73%]) of matched individuals (Fig. 4). However, posthoc tests comparing survivorship between treatments for each sex individually were not statistically significant (Fisher’s exact tests, P = 0.23 for males and P = 0.12 for females). Finally, lizard size (i.e., snout-vent length) did not have a significant effect on survivorship (χ^2^_1_ = 2.94, P = 0.09).

Mass of surviving lizards was significantly lower after trials than it was at the beginning of trials (t-test, t_257.79_ = -5.57, P < 0.01), likely reflecting the effects of food limitation and stress due to the high lizard density in enclosures. However, loss of mass throughout trials was similar for substrate-matched and substrate-mismatched individuals (t-test, t_128.17_ = 0.20, P = 0.84), indicating that lizards in different paint treatments were not differentially affected. We did not observe any instances of tail autotomy throughout the experiment.

Over the two summers that we conducted the study, we found evidence of avian predators (i.e., tracks, scat, or visual observations of the birds themselves) near enclosures on 29 occasions (18 of those being five or fewer meters from the enclosure walls) and within enclosures on nine occasions. In contrast to the ample evidence of avian predator activity, we observed mammal tracks near enclosures on only two occasions, and snake tracks on only one occasion.

## Discussion

We found strong evidence of predation on lizards at White Sands. An average of 36% of lizards survived in the open enclosure treatments during 2011, and 61% survived in 2012. In contrast, 100% of lizards survived in the closed treatments, which were identical to open treatments with the exception that predators were excluded. Additionally, 53% of predator observations occurred at the enclosure with the lowest mean lizard survivorship, which further suggests that lizard mortality was caused by predation in our study.

Avian predators were most likely responsible for the observed mortality in open enclosures. Visually oriented avian predators such as *Geococcyx californianus* (the greater roadrunner) and *Lanius ludovicianus* (the loggerhead shrike) occur at White Sands [14–16], typically hunt during *H. maculata’s* activity period [13, 35–36], and could easily enter and exit the open enclosures. In addition, we frequently observed *L. ludovicianus* in close proximity to enclosures, as well as *G. californianus* tracks, *Corvus cryptoleucus* (Chihuahuan raven) tracks, and small anisodactyl tracks (likely belonging to *Mimus polyglottos* [the northern mockingbird]) within enclosures. In contrast, we observed mammal tracks (likely belonging to *Canis latrans* [coyotes] and *Vulpes marcrotis* [kit foxes]) and snake tracks (likely belonging to *Pituophis catenifer* [Gophersnakes]) near enclosures on only a handful of occasions. We did not find evidence of mammals or reptiles (besides *H. maculata)* within our enclosures at any time throughout the study, indicating that they were not responsible for the majority of the predation we observed.

Although lizards experienced high rates of predation in enclosure trials, survivorship did not differ significantly with respect to paint color treatment. This result is somewhat perplexing, given that previous studies offer strong evidence that blanched color is adaptive in White Sands [17, 23–24, 28]. In a system amenable to experimental manipulation where divergence is literally in black and white, why did we fail to detect selection on color? There are a number of challenges with measuring natural selection in the wild, some of which may have contributed to our inability to detect an effect of color on survivorship. Here we use our results as a case study to examine six major categories of challenges frequently encountered in field-based studies of natural selection, and show that even in seemingly simple systems selection is often complex and dynamic.

### Statistical Power

One challenge with detecting selection in the wild is obtaining a sample size large enough to observe statistically significant results. While statistical power can be an issue in any empirical study, it is particularly problematic in studies of selection in the wild because the strength of selection on morphological traits in natural populations is often quite weak [8, 37–39]. It can therefore be difficult (or even unethical) to attain the sample size necessary to detect the ongoing effects of selection, and consequently many previous studies have focused on species/populations where it is feasible to take a large number of individuals (e.g., [3, 40–44]). To determine whether statistical power could have contributed to the absence of an effect of paint treatment on survival in our enclosure trials, we conducted a power analysis using the effect size of color on survivorship observed throughout the duration of the study. The power analysis indicated that a sample size of greater than 5000 lizards would be required to detect a significant difference in survivorship between substrate-matched and substrate-mismatched lizards. Obtaining such a large sample over the timeframe necessary to carry out the study would not only be unethical, but likely impossible, due to the small size and isolated nature of the White Sands population.

### Sex and Life Stage Variations in Selection

A second category of challenges concerns selection that varies within a species with respect to sex and/or life stage of individuals. Previous studies indicate that the magnitude and direction of selection can vary between sexes and among life stages, with certain traits favored early in life but not later, or in one sex but not the other (e.g., [45–46]). This can have a huge impact on the outcome of studies of local adaptation, where depending on which groups researchers choose to focus on, observed patterns of natural selection could be completely different. In our study, we measured survivorship of adult males and females in enclosures. We found a marginally significant interaction between sex and paint color where substrate matching may have been more important for male survival than female survival. Specifically, males exhibited the expected pattern of higher survivorship of substrate-matched individuals than substrate-mismatched individuals, while in females the opposite was true. The most likely explanation for the interaction effect is that there is a stronger relationship between substrate matching and survival for males than females. Male iguanid lizards are territorial and spend more time during the breeding season being behaviorally conspicuous with territorial and courtship displays [47], and some prior research suggests that conspicuousness can have higher fitness costs for males than females in reptiles [48].

While we might have avoided the potentially confounding effect of variation between the sexes by focusing exclusively on selection in one sex, we did not originally anticipate that color would impact survival of males and females differently. In addition, based on power analyses using the magnitude of the effect of color on survivorship observed in males only, a sample size of approximately 350 males would be required to have a 95% chance of detecting a significant difference (nearly three times the number of males used in our study). We did not repeat the experiment with only males due to the concern that taking 350 males of reproductive age could reduce population size and genetic variation. We cannot evaluate the effect of life stage on survivorship in our study, because we focused exclusively on adults. However, if selection for substrate matching is stronger for juveniles than adults in White Sands, a possible explanation for the absence of a paint treatment effect is that we studied the “wrong” life stage.

### Spatially and Temporally Variable Selection

Yet another challenge with measuring natural selection in the wild is the potential for spatial and temporal variation in selection. While most previous studies do not include spatial or temporal replicates [8, 49], some studies have demonstrated that the magnitude and direction of selection on phenotypes can fluctuate over space (i.e., geographic location) and/or time (e.g., year or season) (e.g., [49–58]). Measuring aspects of fitness over too narrow a spatial or temporal scale can result in an incomplete picture of the impact of natural selection on ecologically relevant phenotypes.

Survivorship in our study varied dramatically with respect to space and time. Year, geographic location, and the interaction between year and geographic location had significant effects on individual survival. It is interesting to note that variation in survivorship occurred over extremely short spatial scales in our study, with significant differences between enclosures separated by a distance of only 110 meters. In addition, survivorship varied drastically from year to year at specific enclosures, with enclosures that exhibited high survivorship in 2011 exhibiting low survivorship in 2012 and vice versa. These patterns of variation could have to do with any number of aspects of the system’s ecology, including biotic and abiotic factors such as predator activity and weather conditions (e.g., [59–60]). For example, predator densities could fluctuate over small spatial scales depending on whether enclosures were located near suitable perches/nesting sites, and predator behavior could fluctuate over small temporal scales depending on whether trials took place during parts of the season where predators were defending territories, breeding, feeding chicks, etc. To investigate the effect of temporal and spatial variation on our ability to detect significant differences in survivorship, we conducted power analyses using the effect size observed within a single enclosure replicate where the magnitude of the effect of color was the strongest. A total of 84 lizards would be required to have a 95% chance of detecting a significant difference, if selection over time and space had remained consistently strong (a sample one third the size of that used in our study). In other words, we would likely have detected an effect of paint treatment if variation between years and among sites had been lower, indicating that spatial and temporal variability had a considerable impact on our study.

### Historical Versus Contemporary Selection

Studies of natural selection in the wild can be complicated by differences between historical and contemporary selection pressures. In addition to variation over small spatial and temporal scales (addressed above), selection pressures may also change over longer time periods, where the magnitude, direction, and/or mode of contemporary selection could be different than past dynamics. Historical selection pressures can have lasting effects on a population. For example, traits that originate in a population to allow individuals to avoid predation can remain widespread even if the dynamics of predation change such that the traits are no longer necessary for survival—a phenomenon termed “the ghost of predation past” (sensu [61]). "Selection past" could in part explain our inability to detect an effect of color on survivorship in White Sands *H. maculata*. It is possible that color was historically more important for survivorship in White Sands, but contemporary selection is less intense due to changes in the ecology of the system. Changes in the abundance of predators of *H. maculata* have occurred in New Mexico in the recent past due to anthropogenic factors. For instance, *L. ludovicianus* has experienced declines across the country since 1966, with some of the highest negative trends occurring in regions of New Mexico [62–63]. If densities of visually oriented predators are drastically different now than they were 50 years ago, we might not detect current differences in survival based on color even though there was historically strong selection for substrate matching. Selection on—or learning by—predators can also alter dynamics of selection on prey species, and could, in theory, result in predators adapted to detect blanched lizards on White Sands. However, the ecotone is narrow relative to the home range size of key avian predators in the region (e.g., *G. californianus* [64]), making it likely that these predators regularly hunt in White Sands, ecotone, and dark soils habitats. We would therefore not expect that habitat-specific specialization in predators has shifted the dynamics of selection on White Sands lizards over time.

### Selection on Correlated Traits

Selection on correlated traits is an additional factor that can complicate studies of natural selection in the wild. When the phenotypic values of multiple traits are correlated as a result of genetic covariances, selection on one trait can have indirect effects on correlated characters [65]. Previous studies have found that correlation between traits can complicate measurement of phenotypic selection, making it difficult to determine whether traits that vary between populations are the direct targets of divergent selection or merely correlated with them [37, 66–67].

In White Sands *H. maculata*, body color is likely important for more than just crypsis; reptiles are ectothermic, and coloration affects an individual’s ability to thermoregulate [68]. However, patterns of body color evolution in White Sands lizards appear to be in the opposite direction of what would be expected if thermoregulatory ability is the primary target of selection. Due to the unique thermal properties of gypsum, the surface temperature of White Sands is much cooler than that of the surrounding desert. Previous research has shown that White Sands *H. maculata* captured in the field exhibit lower body temperature than dark soils individuals [69], and our data demonstrate that painted lizards from matched and mismatched treatments exhibit similar thermoregulatory capabilities on White Sands substrate. It therefore seems unlikely that blanched color confers a fitness advantage to White Sands lizards in terms of thermoregulation- and in fact the opposite may be true. Blanched coloration could potentially make it more difficult for White Sands lizards to achieve and maintain preferred body temperature in the comparatively cool White Sands environment. In addition, the subset of animal species inhabiting White Sands that exhibit blanched coloration represent a variety of divergent taxa (including reptiles, amphibians, mammals, and invertebrates), and certainly do not all possess the same thermoregulatory mechanisms as *H. maculata*. Thus the convergence in color that has occurred among White Sands fauna is likely best explained by the shared necessity of avoiding predation, as opposed to thermoregulatory requirements. The effect of body color evolution on thermoregulation is an important area of future study in the White Sands system.

### Deviations from Natural Conditions

A final challenge in experimental studies of selection is the difficulty of replicating natural conditions in an experimental context. Enclosure experiments often expose individuals to conditions that would not be encountered in nature, and these artificial conditions can have unanticipated effects that make it difficult to accurately measure selection [37]. For example, in our study each enclosure started at a density of 14 lizards per 100 square meters, which is a much higher than the density at which *H. maculata* naturally occur in White Sands (less than one lizard per 100 square meters [13]). The density of conspecifics can affect predation rates experienced by a population [70–71]. It is thus possible that we observed a "buffet effect" in our study, where visually oriented avian predators were initially attracted to the enclosures by substrate-mismatched lizards, but subsequently proceeded to consume lizards of both paint treatments. There was also a limited amount of available ground cover that all lizards within a given enclosure were required to share when seeking shelter from potential predators. It is possible that substrate matching alone is insufficient to avoid predation when escape opportunities are limited, and the abnormally high density of lizards within enclosures could have amplified this effect. The high density of lizards in our enclosures could also have caused undetected changes in behavior associated with increased foraging and competition for limited resources. Similarly, the paint treatment could have caused undetected changes in thermoregulatory behavior of lizards in the enclosures (although we did not observe differences in body temperature or thermal preference in the lab). Thus it is possible that the experimental design had unanticipated effects on other aspects of conspicuousness besides crypsis.

## Conclusion

The challenges listed above are quite common—and we provided evidence that many may have affected our study. Our experiences, along with previously published research, indicate that decisions about the scope of a selection experiment can be particularly influential. For instance, choosing to exclude particular groups (e.g., focusing on only one sex) can result in a misunderstanding of the dynamics of selection. In addition, choice of spatial/temporal scale (e.g., conducting a study over one or multiple years/geographic locations) can lead researchers to completely different conclusions about the direction and magnitude of selection on specific traits. Investigating the effects of “hidden” factors such as sex, life stage, time, and space almost always leads to a more accurate and nuanced picture of selection in wild. Preliminary work that can inform strategic decisions about the scope of an experimental study is particularly important.

Additionally, our study gives insight into the complications associated with experimentally manipulating vertebrates to learn about natural selection in the wild. In particular, experimental manipulation likely had a number of unintended consequences with respect to the behavior of our study organisms including lizard foraging behavior, escape behavior, thermoregulatory behavior, and social interactions. Experimental design could also have affected predator behavior (e.g., if the high density of prey within enclosures led predators to employ different hunting strategies). Experimental manipulation is a key tool for identifying phenotypic targets of natural selection. Complicating factors that arise as a byproduct of manipulating the study organism must be addressed through careful experimental design and data interpretation.

The results of our study indicate that, despite the difficulties detailed above, researchers often learn fascinating things about selection when experiments have unexpected outcomes. For instance, we documented spatial variation in predation over extremely fine scales in the White Sands system. Thus the White Sands system presents an exciting opportunity for future research to investigate scale-specific questions of how and why predation (and potentially selection) varies over space and time. In addition our data indicate that an individual’s sex likely plays a role in determining the effect of body color on survivorship, and thus future research at White Sands will focus on gaining an in-depth understanding of the causes of sex-specific differences in susceptibility to predation. The challenges encountered by our study—and the unexpected results revealed—represent exciting avenues for future research. As evolutionary biologists endeavor to better understand the adaptive significance of specific traits, it is important to consider factors that can generate complex patterns of natural selection even in seemingly simple systems. Studies that assess the effects of these factors on fitness are essential in gaining a nuanced, comprehensive, and accurate understanding of adaptation in the wild.
